# Lung tumor promotion by chromium-containing welding particulate matter in a mouse model

**DOI:** 10.1186/1743-8977-10-45

**Published:** 2013-09-05

**Authors:** Patti C Zeidler-Erdely, Terence G Meighan, Aaron Erdely, Lori A Battelli, Michael L Kashon, Michael Keane, James M Antonini

**Affiliations:** 1 Health Effects Laboratory Division, National Institute for Occupational Safety and Health, 1095 Willowdale Road MS L2015, Morgantown, WV 26505, USA

**Keywords:** A/J mouse, Cancer, Chromium, Nickel, Welding

## Abstract

**Background:**

Epidemiology suggests that occupational exposure to welding particulate matter (PM) may increase lung cancer risk. However, animal studies are lacking to conclusively link welding with an increased risk. PM derived from stainless steel (SS) welding contains carcinogenic metals such as hexavalent chromium and nickel. We hypothesized that welding PM may act as a tumor promoter and increase lung tumor multiplicity *in vivo*. Therefore, the capacity of chromium-containing gas metal arc (GMA)-SS welding PM to promote lung tumors was evaluated using a two-stage (initiation-promotion) model in lung tumor susceptible A/J mice.

**Methods:**

Male mice (n = 28-30/group) were treated either with the initiator 3-methylcholanthrene (MCA;10 μg/g; IP) or vehicle (corn oil) followed by 5 weekly pharyngeal aspirations of GMA-SS (340 or 680 μg/exposure) or PBS. Lung tumors were enumerated at 30 weeks post-initiation.

**Results:**

MCA initiation followed by GMA-SS welding PM exposure promoted tumor multiplicity in both the low (12.1 ± 1.5 tumors/mouse) and high (14.0 ± 1.8 tumors/mouse) exposure groups significantly above MCA/sham (4.77 ± 0.7 tumors/mouse; *p* = 0.0001). Multiplicity was also highly significant (*p* < 0.004) across all individual lung regions of GMA-SS-exposed mice. No exposure effects were found in the corn oil groups at 30 weeks. Histopathology confirmed the gross findings and revealed increased inflammation and a greater number of malignant lesions in the MCA/welding PM-exposed groups.

**Conclusions:**

GMA-SS welding PM acts as a lung tumor promoter *in vivo*. Thus, this study provides animal evidence to support the epidemiological data that show welders have an increased lung cancer risk.

## Introduction

Welding, a process used to join metals, is common in the manufacturing, construction, and other industrial sectors in the U.S. and worldwide. Daily, millions of workers are exposed to welding fume, a complex aerosol mixture of gases and metal-rich particulate matter (PM). Because of the presence of known human carcinogens, such as hexavalent chromium (Cr(VI)) and nickel (Ni), the potential carcinogenicity of welding fume is a critical concern in occupational toxicology. In fact, the International Agency for Research on Cancer (IARC) advisory group on the *Monograph* priorities for 2010–2014 listed welding fume as a high priority agent for further evaluation of carcinogenic risk to humans [1]. Currently, the IARC classifies welding fumes as a group 2B carcinogen (possibly carcinogenic to humans); however, this categorization was based on limited epidemiology and inadequate animal data [2-4].

Occupational exposure to welding fume is unique and presents numerous physical (e.g. heat and UV radiation) and chemical hazards to the worker. Mixtures of metal compounds of iron (Fe), Cr(VI), manganese (Mn), Ni, and gases (e.g., ozone, carbon monoxide, nitrogen oxides) all may be present in the fume [5,6]. In addition, the fume is classified as an incidental nanoparticle as significant numbers of ultrafine (< 0.1 μm) particles are formed during the welding process [7]. As such, both respiratory and non-respiratory adverse health effects are well-documented in workers and can include bronchitis, immunosuppression, pneumonia, metal fume fever, siderosis, and neurological effects [5,8].

Fume generated during gas metal arc (GMA)-stainless steel (SS) welding is largely water-insoluble and closely resembles the metal composition of the consumable electrode wire used [5]. Cr(VI) and Ni are present in significant amounts in this fume and are necessary for corrosion protection of the weld [9]. Previous experimental evidence showed that welding PM, from SS welding wire in particular, caused epithelial injury as well as atypical and hyperplastic cellular changes in the lungs of mice. Interestingly, a mild chronic lung inflammation was accompanied by an increased persistence of these fumes *in situ*[10,11]. In addition, evidence for a weak (borderline significant) carcinogenic effect in lung tumor susceptible mice was found for PM from GMA-SS welding, one of the most prevalent workplace processes [11]. Predicated on these findings and suggestions by the IARC, this current study is a continuation of previous investigations by our laboratory to evaluate the carcinogenic potential of welding fume. Here, we focus on carcinogenic metal-containing SS welding PM as a tumor promoter using a two-stage (initiation-promotion) mouse lung tumor bioassay.

## Results

### Welding fume PM Cr(VI) and total metal analysis

GMA-SS welding PM contained the following metals (weight %): Cr (20.2), copper [Cu] (0.2), iron [Fe] (57), Mn (13.8) and Ni (8.8) with trace amounts of silicon, aluminum, and vanadium. Cr(VI) levels in the fume were 2929 ppm (μg/g) (n = 3, SD = 120).

### Morbidity and mortality

**Figure 1:**
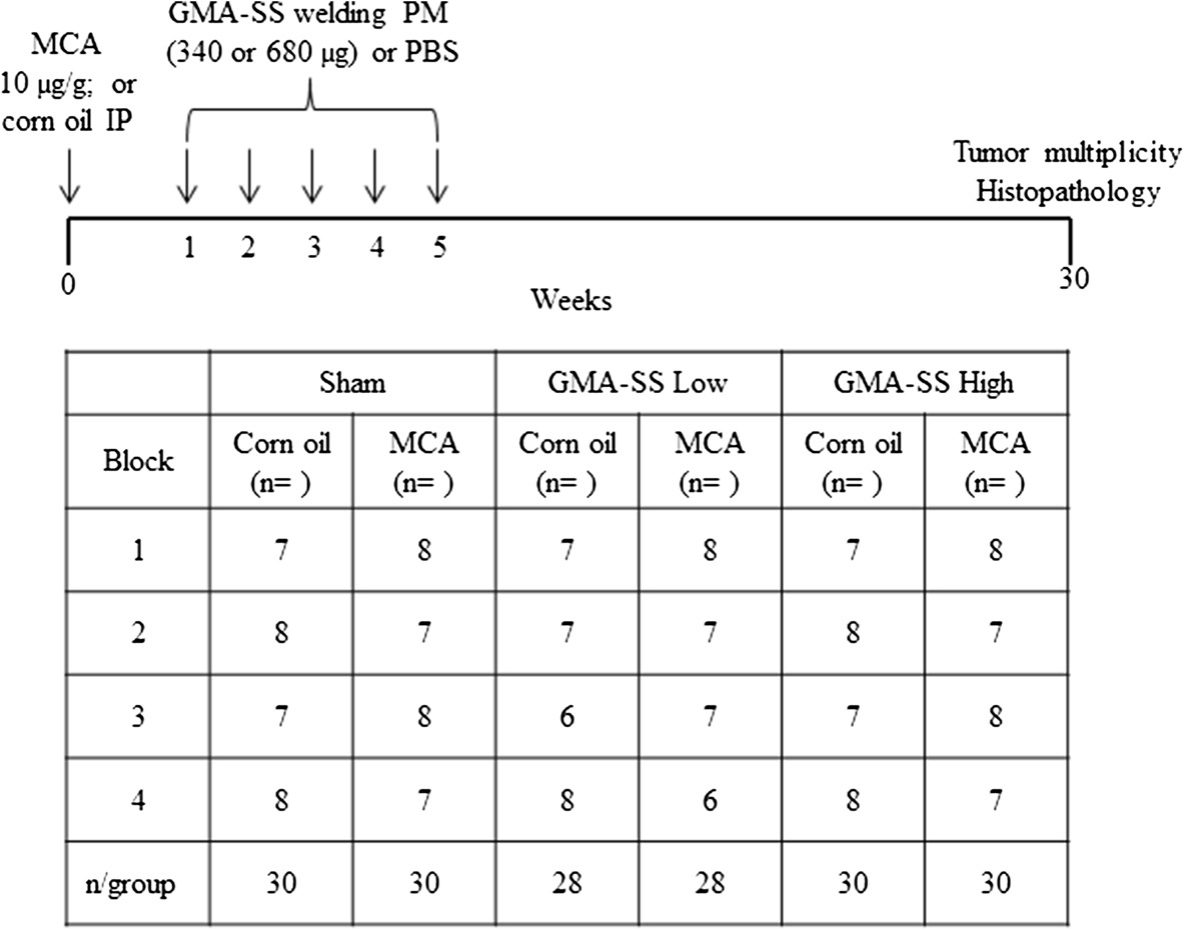
**Experimental protocol and block design for the two-stage carcinogenesis model to assess GMA-SS welding PM as a tumor promoter in A/J mice.** Mice were IP injected with MCA (initiator) or corn oil (vehicle) then 1 week later exposed to GMA-SS (340 or 680 μg) or PBS (vehicle; sham) by pharyngeal aspiration once a week for 5 weeks. The study was carried out in four blocks (1 block/day for 4 days/week) for randomization. All treatment combinations were represented in each block. Body weights were recorded at week 0, then at each weekly aspiration exposure and at every two weeks thereafter. Mice were sacrificed 30 weeks post-initiation and tumor multiplicity was evaluated.

A timeline of the experimental protocol for the two-stage (initiation-promotion) carcinogenesis model is shown in Figure 1 and described in detail in the methods section. Body weights were recorded at 2 week intervals throughout the study and no effect of exposure was found. Body weight changes from week 0 to 30 were (mean ± standard error [SE]) 6.83 ± 0.27, 7.19 ± 0.37, and 6.41 ± 0.37 for the corn oil/sham, GMA-SS low and high groups, respectively. For the MCA/sham, GMA-SS low and high groups body weight changes were 7.30 ± 0.36, 7.36 ± 0.39 and 7.23 ± 0.31, respectively. Morbidity and mortality throughout the study was low and no abnormalities, such as other tumor types besides lung, were found at the terminal sacrifice at 30 weeks. In total, 13 mice died during the course of the study (~93% survival rate) and were not included in the final analysis of the data. Of the >880 pharyngeal aspirations performed for the study, 4 mice died from effects likely related to anesthesia during the exposures. Nine mice died with typical morbidities including head tilt, scrotal or mesenteric abscesses, kidney masses, enlarged hearts or from unknown causes. This indicated that the experimental protocol was well tolerated.

### Gross tumor multiplicity and incidence

**Figure 2:**
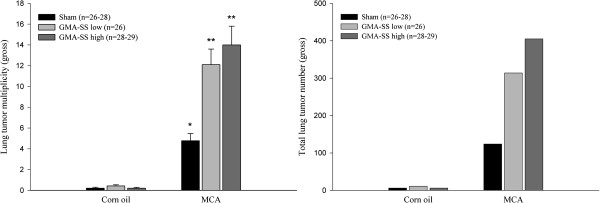
**Lung tumor multiplicity (left panel) and total lung tumor number (right panel) upon gross examination in A/J mice promoted with PBS (sham) or GMA-SS welding PM.** At 30 weeks, MCA initiation followed by GMA-SS exposure increased lung tumor multiplicity (average number of tumors/mouse ± SE) in both the low dose (12.1 ± 1.5) and high dose (14.0 ± 1.8) groups significantly above that of MCA/sham (4.77 ± 0.7). **p < 0.0001; *p < 0.0001- compared to corn oil/sham. Right panel shows increased total lung tumor numbers in the MCA/GMA-SS low and high dose groups above that of the MCA/sham.

In the presence of MCA, GMA-SS welding PM was a highly significant promoter of lung tumor number in the A/J mouse 30 weeks after initiation. Figure 2 shows the grossly observed tumor multiplicity (average tumor number/mouse lung ± SE) (left panel) and the total tumor numbers (right panel) for all groups. Tumor multiplicity in the low and high dose group was 12.1 ± 1.5 (*p* = 0.0001) and 14.0 ± 1.8 (*p* = 0.0001), respectively, compared to MCA/sham 4.77 ± 0.7 Multiplicity was also highly significant across all five individual lung regions of GMA-SS-exposed mice (*p* < 0.004) (Table 1). In the corn oil groups, tumor multiplicity was 0.21 ± 0.09, 0.42 ± 0.11, 0.21 ± 0.08 in the sham, GMA-SS low and high groups, respectively, and was not significant across exposure groups. No significant difference in multiplicity was found between the low and high dose GMA-SS groups initiated with corn oil or MCA. Average tumor incidence (% of tumor-bearing mice) was 25.8 ± 6.4% and was not significantly different across exposure groups treated with corn oil. As expected, incidence was >93% in all MCA-initiated groups which verified successful experimental administration as well its carcinogenic effectiveness in A/J mice.

**Table 1:** Total tumor number across individual lung lobes in A/J mice following exposure to GMA-SS welding PM at 30 weeks post-initiation with MCA or corn oil

	**n**	**Left**	**Apical**	**Cardiac**	**Diaphragmatic**	**Azygos**
Corn oil/Sham	28	1	1	2	2	0
Corn oil/GMA-SS low	26	4	3	2	1	1
Corn oil/GMA-SS high	28	4	1	0	1	0
MCA/Sham	26	52^*^	12^*^	13^*^	39^*^	8^*^
MCA/GMA-SS low	26	119^**^	46^**^	40^#^	81^#^	28^#^
MCA/GMA-SS high	29	132^**^	64^**^	66^**^	106^**^	37^#^

*GMA-SS* gas metal arc-stainless steel, *PM* particulate matter, *MCA* 3-methylcholanthrene.

^*^p < 0.002 - compared to corn oil/sham.

^**^p < 0.0007, ^#^*p* < 0.004 - compared to MCA/sham.

**Figure 3:**
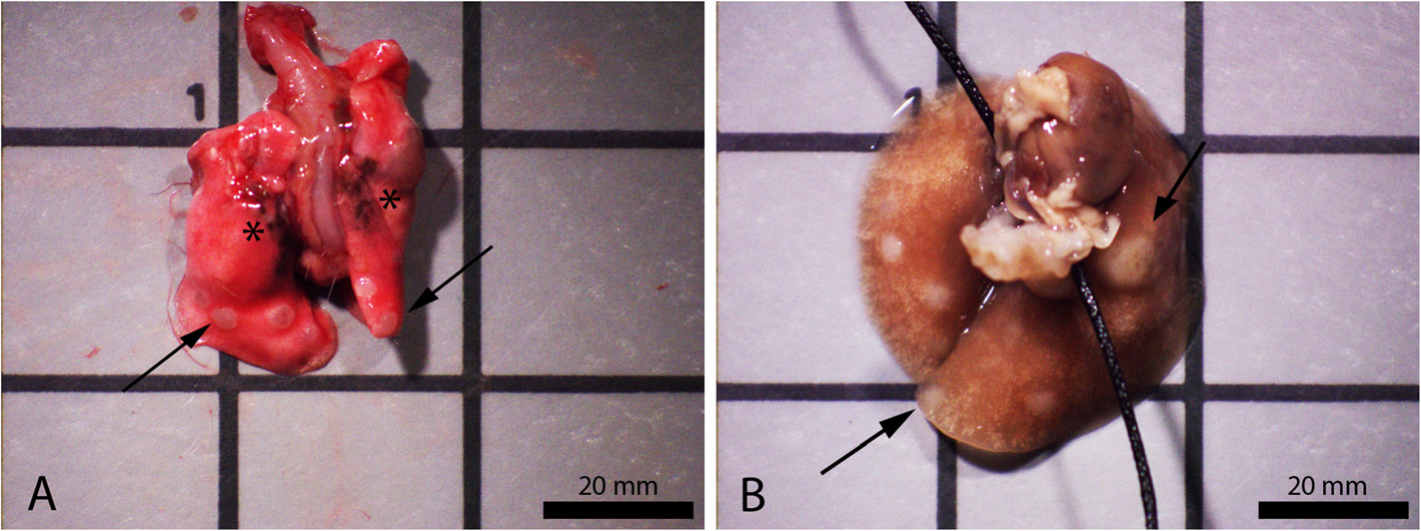
**Gross images of lung tumors promoted by GMA-SS welding PM 30 weeks after initiation with MCA.** Panel **A** represents the lung tumor morphology before fixation panel **B** is 24 h after fixation. Asterisks (*)-indicate areas of welding PM deposition. The arrows (↑)-indicate lung tumors. The majority of tumors were ~1 mm in diameter.

Gross lung morphology from a welding PM-exposed mouse initiated with MCA is shown in Figure 3. Tumors (arrows) appeared white in color and semi-translucent to opaque upon initial gross exam (A). After fixation, tumors were more defined which facilitated enumeration (B). At 30 weeks, all tumors were ≥1 mm and ≤4 mm in diameter; however, the majority were ~1 mm. Welding PM (*) was found in all exposed mouse lungs and appeared dark brown to black in color.

### Histopathological evaluation of lung lesions, inflammation, and welding PM presence

**Figure 4:**
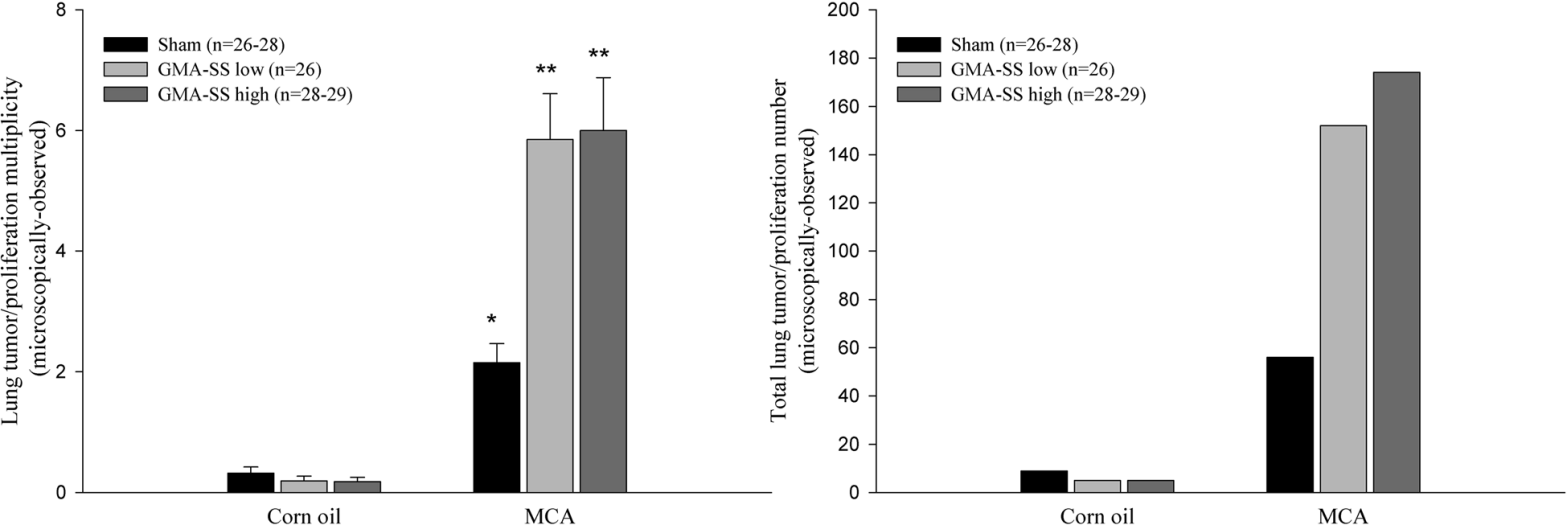
**Lung tumor/proliferation multiplicity (left panel) and total number (right panel) microscopically observed in A/J mice promoted with PBS (sham) or GMA-SS welding PM.** At 30 weeks, MCA initiation followed by GMA-SS exposure increased multiplicity (average number of tumors and proliferations/mouse ± SE) in both the low dose (5.85 ± 0.76) and high dose (6.00 ± 0.87) groups significantly above that of MCA/sham (2.15 ± 0.32). **p < 0.0001; *p < 0.0001- compared to corn oil/sham. Right panel shows increased total lung lesion numbers in the MCA/GMA-SS low and high dose groups above that of the MCA/sham.

**Figure 5:**
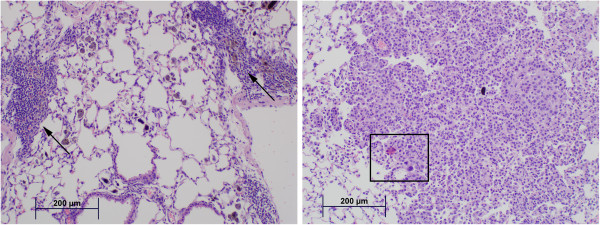
**Photomicrographs of lung tissue from MCA-treated mice.** Representative photomicrographs were captured at 30 weeks after MCA-initiation and show presence of lymphoid infiltrates (i.e., inflammatory cell infiltrates consisting of peribronchial/perivascular associated lymphocytes, macrophages, and plasma cells) and welding PM (arrows) in the MCA/low GMA-SS group (left panel). An area of malignant transformation (rectangle)—larger cells with irregular nuclei and associations—in the MCA/high GMA-SS group (right panel).

Figure 4 shows the histopathological assessment (including preneoplastic epithelial proliferations) for multiplicity (left panel) and the total numbers of lung lesions (right panel) from all groups. Histopathology confirmed the gross results and showed a remarkable similarity between the ratios for gross tumor counts and those enumerated microscopically (see Figure 2). Tumor incidence was not different among the corn oil- or MCA-treated groups and was, as expected, lower than that obtained by gross exam, 21.9 ± 3.4% and 85.0 ± 4.1%, respectively. Inflammation and welding PM severity scores and number of each lung lesion type are shown in Table 2. Welding PM was found in all exposed lungs and detected in slightly greater amounts in the high dose groups. Lymphoid cell infiltrates, an indicator of inflammation, consisted of peribronchial/perivascular associated lymphocytes, macrophages, and plasma cells and were increased in all welding PM-exposed groups (Figure 5, left panel). Of note, there was no significant difference in inflammatory cell infiltrates in the sham-exposed mice treated with MCA or corn oil 30 weeks after IP injection. This dose of MCA has also been shown to be non-inflammatory by bronchoalveolar lavage analysis in the A/J mouse [12]. Microscopically, adenomas and preneoplastic epithelial proliferations were in the majority, which is consistent with the model and previous observations [11,13]. There were a greater number of malignant lesions in the MCA/GMA-SS high group compared to MCA/sham; 7 out of 29 mice were found to have a CA or C (Figure 5, right panel). In the MCA/GMA-SS low group, there were 2 animals with advanced lesions, one with a multiple CA, so the significance in this case should likely be dismissed.

**Table 2:** Severity scores for abnormal morphological findings and number of microscopically observed lung lesion types in A/J mice following exposure to GMA-SS welding fume PM at 30 weeks post-initiation with MCA or corn oil

	**Lymphoid**	**Welding-fume**	**Preneoplasia**	**Adenoma within**	**Adenoma**	**Adenocarcinoma**	**Carcinoma**
	infiltrates^*^	laden cells^*^		**preneoplasia**			
Corn oil/Sham	0.25 ± 0.07	0.00 ± 0.00	5	2	2	0	0
Corn oil/GMA-SS low	1.79 ± 0.07^**^	2.12 ± 0.04	1	0	3	1	0
Corn oil/GMA-SS high	1.84 ± 0.06^**^	2.29 ± 0.07^#^	0	1	4	0	0
MCA/Sham	0.15 ± 0.00	0.00 ± 0.00	16	5	34	0	1
MCA/GMA-SS low	1.44 ± 0.08^**^	1.90 ± 0.05	61^**^	17^**^	70^**^	4^**^	0
MCA/GMA-SS high	1.57 ± 0.06^**^	2.12 ± 0.06^#^	65^**^	9	93^**^	6^**^	1

*GMA-SS* Gas metal arc-stainless steel, *PM* Particulate matter, *MCA* 3-methylcholanthrene.

^*^Severity scores were average of the right and left lung lobe score and are presented as mean ± standard error, lymphoid infiltrates are peribronchial/perivascular associated lymphocytes, macrophages, and plasma cells. Severity was scored as: 1 = minimal, 2 = mild, 3 = moderate, 4 = marked, 5 = severe.

^**^p < 0.01 - compared to the corresponding sham in the corn oil- or MCA-treated groups.

^#^*p* < 0.001 - compared to the corresponding GMA-SS low in the corn oil- or MCA-treated groups.

## Discussion

The novel finding of this study was a highly significant increase in lung tumor multiplicity in mice promoted with SS welding PM, which was consistent and significant across all five individual lung regions. This response was observed 30 weeks after MCA-initiation. Also of note, there were more malignant lesion types in the MCA/high dose welding PM group which suggests that not only is the rate of tumor formation being increased by welding PM, but the progression to malignancy appears to be affected with higher doses of particulate. This study is the first to link enhanced lung tumor formation and welding fume exposure *in vivo* and provide animal evidence to support epidemiological findings.

A 25% to 40% increased risk of lung cancer has been associated with the welding occupation [14-16]. Indeed, the proportionate mortality ratio for welders for lung cancer is 1.2 [17]. Even though some evidence exists to the contrary, epidemiological studies generally support an increased risk, but they are limited in number; animal studies are scarce [3]. Because welders work under widely diverse conditions and co-exposures such as silica, smoking, and asbestos may be involved, cumulative exposure data and a complete occupational history may not always be available [15,18,19]. Therefore, controlled animal studies to elucidate the underlying factors of welding fume-related lung carcinogenesis are long overdue.

Previously, we assessed the ability of different types of welding PM to act as a complete lung carcinogen in lung tumor susceptible A/J mice. Efforts of those studies were ultimately negative but hinted at a potential weak carcinogenic effect of SS welding PM, as a borderline significant (p = 0.057) increase in grossly observed lung tumor incidence (i.e., presence or absence of tumors) was found [10,11,20,21]. In addition, histopathology at 78 weeks after exposure revealed presence of SS welding PM which was associated with a mild, but significant, chronic inflammatory cell influx in the lung tissue. Of note, these effects were not observed following exposure to mild steel (MS) welding PM composed largely of iron oxide [11]. Also, to complement those studies, the lung toxicity and gene expression profiles in the tumor susceptible A/J and resistant C57BL/6J (B6) mouse were compared following pharyngeal aspiration of GMA welding PM [11,22]. Interestingly, a significantly greater magnitude of overt lung toxicity (polymorphonuclear leukocyte influx, lung cytotoxicity and permeability) and an attenuated resolution of the inflammatory response to different types of welding PM were found in the A/J versus the B6 mouse strain. Results from the microarray analysis confirmed those aforementioned responses and revealed a greater lung transcriptional gene activation as well as a prolonged dysregulation of immunomodulatory genes after welding PM exposure in the A/J versus the B6 mouse [22]. In all cases, the lung toxicity and transcriptional effects were greater with the carcinogenic metal-containing SS welding fume when directly compared to a MS welding fume. Therefore, historical data in our laboratory suggested that fumes containing Cr and Ni were the most toxic, persisted in the lung longer as compared to other types, and were possibly tumorigenic *in vivo*.

The A/J mouse lung tumor bioassay is well-characterized, has good inter- and intra-laboratory reproducibility, and has been widely used for testing hundreds of potential lung carcinogens, such as tobacco smoke and polycyclic hydrocarbons [23-25]. It also continues to be useful for evaluation of chemointerventive agents of lung neoplasia [24,26,27]. This strain is susceptible to both chemically-induced and spontaneous lung adenomas compared to the resistant B6 mouse [23]. Morphological, molecular, and histological features of the lung tumors that arise in these mice resemble human adenocarcinomas; therefore, findings in this model have direct human relevance [28]. Indeed, tumor susceptibility in the A/J strain has been associated with a polymorphism in intron 2 of *Kras* and this finding is pertinent to human lung adenocarcinoma development because ~35% of these human tumor types contain *Kras* oncogenes [29,30].

In humans, chronic lung inflammatory conditions such as asthma and chronic obstructive pulmonary disease are associated with increased risk of lung cancer and epidemiology suggests ~25% of human cancers are attributed to chronic inflammation [31-33]. Microenvironments of chronic lung inflammation, largely dominated by macrophages and other leukocytes, create a milieu rich in reactive oxygen species and cytokines that may promote tumorigenesis [34-36]. Indeed, in the mouse model, quantitative trait loci (QTL) that control genetic susceptibility to lung inflammation colocalize with tumor susceptibility QTL [37]. Two-stage carcinogenesis in lung tissue was first reported by Witschi et al. when repeated IP injections of butylated hydroxytoluene (BHT), a synthetic food additive and antioxidant, increased lung adenoma multiplicity in both Swiss-Webster and A/J mice initiated with a single dose of the potent carcinogen urethane 9–15 weeks prior [38]. The model of BHT tumor promotion continues to provide mechanistic insight into the critical role that inflammation has in lung tumor initiation and promotion [35,39,40]. More recently, this two-stage model was used to demonstrate the *in vivo* promoter activity of vanadium pentoxide (V_2_O_5_), a component of environmental and occupational PM, in A/J, BALB/cJ, and C57BL/6 J mice initiated with MCA 20 weeks prior [12]. In agreement with our earlier findings that strain-dependent (A/J > B6) lung responses were evident after welding PM exposure, Rondini et al. found that V_2_O_5_-mediated lung inflammation and subsequent tumor multiplicity also showed strain dependency (A/J > BALB/cJ > B6) [11,12,22]. In this study, the observed inflammatory cell infiltration and highly significant increased tumor multiplicity after MCA/GMA-SS welding PM exposure, combined with our previous evidence of chronic inflammation due to SS particle persistence in the lung, further supports the role of inflammation in the promotion of lung tumors in A/J mice.

GMA-SS welding PM is poorly soluble and contains toxic metals, namely Cr(VI), which is carcinogenic, especially in the particulate form [3]. *In vitro*, this fume has also been shown to cause greater DNA damage, lipid peroxidation, and radical generation compared to MS fume [41]. Increased DNA damage has also been reported in blood leukocytes from welders exposed to Cr and Ni fumes [42,43]. Once inhaled, Cr(VI) particles are retained primarily by the lung and tend to accumulate near major bifurcations, where they may persist for as long as twenty years [44,45]. A possible mechanism for Cr(VI) carcinogenicity involves the slow release over time of chromate ions from particulate compounds adhered to the cell surface. These ions may escape extracellular reduction by ascorbate, which then allows for uptake by lung epithelial cells causing tumor formation [46]. Indeed, a concentration-dependent induction of aneuploidy has been shown in normal human bronchial fibroblast and immortalized human bronchial epithelial cells exposed to particulate chromate [47,48]. In addition, Cr(VI) may act synergistically with Ni, present in lower amounts in this fume, as suggested by co-mutagenicity studies [49]. In rats and mice, GMA-SS fume exhibited a slower lung clearance timeline compared to a more soluble manual metal arc-SS (MMA-SS) and GMA-MS fume [11,50]. Thus, slower lung clearance, together with the greater lung toxicity profile of this fume *in vivo*, may significantly contribute to its increased tumor promoter activity.

No threshold limit value-time weighted average (TLV-TWA) exists for welding fume. The previous TLV-TWA of 5 mg/m^3^ for welding fume was retracted in 2004 by the American Conference of Governmental Industrial Hygienists [51]. In this study, we used welding PM exposures equivalent to 1.84 and 3.67 years of work exposures at 5 mg/m^3^ and Cr(VI) exposures of 5.4 and 10.8 years at 5 μg/m^3^ in a human for the low and high doses, respectively. Previously published reports have indicated that airborne concentrations of Cr(VI) in industries using SS welding can be 50–400 μg/m^3^[52]. Welders oftentimes work in confined spaces which can increase the total fume exposure to > 20 mg/m^3^[53]. Because freshly generated welding fume induces greater lung inflammation than “aged” fume, such as that used in this study, and one-third of the dose by inhalation results in about 2 to 3 times the pulmonary toxicity, the exposures used herein are reasonable [11,21,54]. However, the bolus delivery of the particles is an obvious limitation in this study, even though the doses were repeated over a five- week time frame (1 exposure/week). As such, welding fume by inhalation is 6 to 9 times more potent than by pharyngeal aspiration [55]. The mechanisms of increased toxicity by inhalation are likely related to the free radical generation of freshly generated welding fume compared to “aged” fumes that are collected onto filters then used in instillation studies [41,54]. Given the role of inflammation in tumor promotion described above, the combined interpretation of our previous studies strongly suggests that a significantly lower mass deposition by inhalation would have similar results as those in this study.

## Conclusions

The current research supports epidemiological findings that SS welding fume may have carcinogenic potential. At 30 weeks after MCA-initiation, an average of 7.33 to 9.23 tumors/mouse were grossly enumerated after exposure to GMA-SS welding PM. Indeed, the effect was highly significant (p < 0.0001). GMA-SS welding PM did not increase multiplicity or incidence by 30 weeks in the corn oil-treated A/J mouse. Therefore, in this experimental model, this fume does not appear to be a potent initiator and shorten the timeframe for induction of lung tumors, which agrees with previous results [11,21]. Taken together, the intrinsic toxicity of GMA-SS welding PM, the chronic lung inflammatory milieu, and the leaching of Cr(VI) from the particles may allow for potential tumor promotion in a susceptible population.

## Methods

### Animals

Male A/J mice, age 5–6 weeks, were purchased from Jackson Laboratories (Bar Harbor, ME) and housed in an AAALAC-accredited, specific pathogen-free, environmentally controlled facility. All mice were free of endogenous viral pathogens, parasites, mycoplasmas, *Helicobacter*, and CAR Bacillus. Mice were individually housed in ventilated cages and provided HEPA-filtered air under a controlled light cycle (12 h light/12 h dark). Animals were acclimated to the animal facility for a minimum of 1 week and allowed access to a conventional diet (6% Irradiated NIH-31 Diet, Harlan Teklad, Madison, WI) and tap water *ad libitum*. All procedures were performed using protocols approved by the National Institute for Occupational Safety and Health Institutional (NIOSH) Animal Care and Use Committee.

### Generation of GMA-SS welding PM and analysis of Cr(VI) levels

Welding fumes were generated for this study by the NIOSH robotic welding system which includes a 6-axis robotic arm, power supply, water-cooled arc welding torch, and a wire feeder; as previously detailed [7]. SS welding wire was Lincoln Electric E308 LSi; the shield gas was Ar/CO_2_ 95%/5% at 19 liters/min. The welding material in the baseplates was ¼ inch A-36 carbon steel. Welding was done in axial spray mode at 26.5 volts, 240 amperes, 325 inches/min wire feed, and 15 inches/min travel.

Fumes from the weld area were sampled onto electrostatic medium filters (PE 13060NA, Hollingsworth and Vose, East Walpole, MA), and PM was recovered by gentle suction, then ground by shaking for 30 sec in a Wig-L-Bug grinder using a metal-free disposable polyethylene vial with two 1/8 inch silicon nitride-coated ceramic balls. The ground material was anti-static treated, and 5 mg samples were weighed into 15 ml polycarbonate centrifuge tubes.

Three replicate welding PM samples were analyzed for Cr(VI) levels using NIOSH method 7605 [56]. Briefly, 5 ml of extraction solution (3% Na_2_CO_3_/2% NaOH) were added to each 5 mg sample, and the tubes were sonicated in a bath for 30 min. This procedure extracts both soluble and insoluble Cr(VI) present in the fumes. Analysis used a Dionex HPIC-AS7 column with 250 mM (NH_4_)_2_SO_4_/100 mM NH_4_OH mobile phase and a postcolumn reagent (2.0 mM diphenylcarbazide/10% methanol/1 N H_2_SO_4_) with absorbance detection at 540 nm. Four concentrations of standards were made from a certified Cr(VI) solution, covering a range of 0.4-4 μg/ml. The estimated limit of detection is 0.02 μg, and the method range is 0.05 to 20 μg of Cr(VI). Total metal analysis was done as previously described using inductively coupled plasma-atomic emission spectroscopy (ICP-AES) using NIOSH method 7300 modified for microwave digestion [7,57]. The metal solubility of this fume is low (0.006 soluble:insoluble) and the mass median aerodynamic diameter of the sample is 0.255 μm, as previously determined [58].

### Experimental protocols for animal exposures and sacrifice

A/J mice, 176 in total, were organized into 6 groups using a block design for randomization (Figure 1). During week 1, mice were intraperitoneally (IP) injected with an initiator, 3-methylcholanthrene (MCA), (Sigma, St. Louis, MO) dissolved in corn oil (Sigma, St. Louis, MO) at a dose of 10 μg/g body weight or corn oil alone. One week post-initiation, mice were exposed to GMA-SS welding PM suspended in phosphate-buffered saline (PBS) without calcium and magnesium by pharyngeal aspiration as previously described [59]. Briefly, each mouse was placed in a bell jar with gauze moistened with isoflurane (Abbott Laboratories, Abbott Park, IL) until slowed breathing was observed. The mouse was then suspended by its top incisors, on a slanted board in a supine position. The tongue was extended with forceps and the test suspension was placed by pipette at the back of the throat. The tongue was held until the solution was aspirated into the lung and 3 deep breaths were observed. The mouse recovered fully in its cage within ~15 sec. Mice were exposed once a week for 5 weeks to either 340 or 680 μg of freshly prepared GMA-SS welding PM or PBS (sham control). Welding PM was briefly sonicated (< 1 min) after suspension in PBS, then vortexed immediately before each individual animal exposure.

The cumulative exposures over the 5 week time course were 1.7 mg and 3.4 mg, respectively, and these were chosen based on previous published results [10,11,20]. Previous research has estimated that a welder exposed to 5 mg/m^3^ for an 8 h day would be expected to have a lung burden of 7.7. mg alveolar deposition [60]. Utilizing 102 m^2^ for human and 0.05 m^2^ murine alveolar surface area [61], 7.7 mg is converted to a murine equivalence of 3.77 μg. Assuming 100% alveolar deposition of the aspirate, the low dose of GMA-SS PM used in this study would be equivalent to 450 days, or 1.84 working years, of a worker being exposed at 5 mg/m^3^ for 8 h/d. The high dose would be equivalent to 900 days, or 3.68 working years. The current PEL for Cr(VI) is 5 μg/m^3^ which was readjusted in 2006 from 52 μg/m^3^[62]. Therefore, a human exposed to 5 μg/m^3^ Cr(VI) for 8 h would result in 7.7 μg alveolar deposition with a murine daily equivalence of 3.77 ng. In this study, Cr(VI) in the welding PM was measured to be 2920 μg/g, or 0.29%. Thus, the low and high doses contained 4.96 μg and 9.93 μg of Cr(VI), respectively. An exposure level of 5 μg/m3 Cr(VI) for 8 h/d, would be equivalent to 5.4 years for the low and 10.8 years for the high dose.

One block/day (4 days/week) was completed for each stage of the 6-week protocol. MCA was chosen as the initiating agent based on the efficient response of the A/J mouse to this carcinogen compared to other mouse strains [63]. The dose of MCA and basic protocol in A/J mice were derived from a study by Rondini et al. [12].

At 30 weeks post-initiation, mice were sacrificed over a 4 day period (1 block/day). Mice were anesthetized with an IP overdose of Sleepaway (26% sodium pentobarbital, 7.8% isopropyl alcohol and 20.7% propylene glycol, Fort Dodge Animal Health, Fort Dodge, IA) then weighed. Once unresponsive, the abdomen and thoracic cavity were opened and examined for any abnormalities and the vena cava was cut to exsanguinate the mouse. The whole lung was excised then inflated and fixed with 10% neutral buffered formalin for 24 h. Lungs were then examined and tumors were enumerated and measured in a blinded fashion by two individuals with the aid of a Leica MZ6 stereomicroscope (Leica Microsystems, Inc., Buffalo Grove, IL). Apparent merged tumors, defined as a single tumor pattern in double-nodule form or an apparent collision of two different tumors, were counted as one. Gross images were taken using an Olympus DP21 digital camera (Olympus America, San Jose, CA).

Whole lungs were embedded in paraffin then a 5 μm standardized section was cut. Slides were stained with hematoxylin and eosin and interpreted by a contracted board certified veterinary pathologist in a blinded fashion for morphological changes and proliferative/neoplastic lesions. All lung lobes were evaluated from every animal in each group. If abnormal changes were found, severity was scored as follows: 1 = minimal, 2 = mild, 3 = moderate, 4 = marked, 5 = severe. The final severity score reflects the average of the right and left lung lobe scores (see Table 2). Proliferative/neoplastic changes were counted and identified as P = preneoplastic epithelial proliferation, AP = adenoma arising within a proliferation, A = adenoma, CA = carcinoma arising within an adenoma, C = carcinoma, or MC = microcarcinoma [64]. Since examination of a single histological section per lung underestimates the total number of lesions per lung, the gross count at necropsy would be more representative of the response. However, for completeness, both microscopic and gross exam total lung tumor numbers were statistically evaluated in this study.

### Statistics

All analyses were performed using SAS/STAT version 9.3 (SAS Institute Inc., Cary, NC) for Windows. Binary outcomes of tumor incidence (presence or absence) for each region and for the total were analyzed using Fishers Exact test. Tumor counts were analyzed using negative binomial regression for each region and the total because the Poisson regression on counts was overdispersed. All analyses were stratified by promoter. Only animals surviving the entire 30 week time course were used for analysis.

## Abbreviations

B6: C57BL/6 J; Cr(VI): Hexavalent chromium; Fe: Iron; GMA-SS: Gas metal arc-Stainless steel; GMA-MS: Gas metal arc-mild steel; IARC: International agency for research on cancer; IP: Intraperitoneal; LOD: Limit of detection; Mn: Manganese; MMA-SS: Manual metal arc-stainless steel; MCA: 3-methylcholanthrene; Ni: Nickel; NIOSH: National institute for occupational safety and health; PM: Particulate matter; PEL: Permissible exposure limit; PBS: Phosphate buffered saline; SE: Standard error; TLV-TWA: Threshold limit value-time weighted average.

## Competing interest

The authors declare that they have no competing interest.

## Authors’ contributions

PCZE and JMA conceived and designed the study. PCZE drafted the manuscript. PCZE, TGM, AE and LAB performed the animal exposures, animal sacrifices, counted the lung tumors, and assisted with lung preparation for histopathology. MK generated the welding fume sample for the exposure and MLK statistically analyzed all data. All authors read and approved the final manuscript.
